# Medical Errors, Regrettable Mistakes in Public Health

**Published:** 2018-07

**Authors:** Shaohan WU, Haikuan WANG

**Affiliations:** School of Life Sciences, Peking University, Haidian District, Beijing, China

## Dear Editor-in-Chief

At the beginning of 2017, a medical error shocked the China society. On Jan 26th, Health and Family Planning Commission of Zhejiang Province in China received a report which said that because of the non-standard medical operation, five patients had got cross-infection of HIV in a Chinese Medicine Hospital in Hangzhou City. Moreover, other patients who involved in this treatment were also at risk of HIV infection.

“Medical error may be defined as any problems in the process of medical services, or failure of a planned health care action or utilizing from an incorrect healthcare action plan to overcome a health problem”. “Or it can be defined as implementations resulting in death, or permanent or temporary damage in the vital functions of a patient” (1). Medication errors can also add a financial burden to the institution as well. “Though the impact varies from no harm to serious adverse effects including death, it needs attention on priority basis since medication errors’ are preventable” (2).

Why could a medical error happen? First of all, workloads may cause a series of careless; also some subjective or objective standard-violated operation can be done for a “time-saving”. Secondly, insufficient education may contribute to irresponsibility. Medical-related staffs may have no idea what is the result of regulations violation. In the former case, the staff did not obey the rule of “one blood collection device for one person” which caused an irreparable disaster in those families. The last cause accounts for medical errors is the vague instruction of a guideline. A misunderstanding in drug usage, operation process, attention in treatment is possible.

Medical care should be the guardian for the public; however, medical errors in the process kill people’s life. Medical errors are uncommon everywhere in the world and may account for as many as 251000 deaths annually in the United States (3). In addition, it has been considered the third leading cause of death in the US (4). Countries and organizations should combine to combat medical errors, and try our best to find out why and how to reduce its happening. Only in this way can our public health system can benefit our world and without regrets.
